# The Effects of Androstenone on the Plasma Serotonin, β-Endorphin, and Cortisol Concentrations in Thoroughbred Horses

**DOI:** 10.3390/ani11061694

**Published:** 2021-06-07

**Authors:** Yeonju Choi, Minjung Yoon

**Affiliations:** Research Center for Horse Industry, Department of Animal Science and Biotechnology, Kyungpook National University, Sangju 37224, Korea; yjchoi2031@gmail.com

**Keywords:** androstenone, horse, neurotransmitter, pheromone

## Abstract

**Simple Summary:**

The development of horse managing tools is needed to prevent accidents and to improve welfare for domestic horses because the safety hazards for people who are exposed to horses are well documented. Androstenone, a pheromone secreted from boars, changes the behavior of dogs to become less excited. In horses, a previous study showed that a specific receptor for androstenone was expressed in the vomeronasal organ and nasal cavity. Horses treated with androstenone also showed more compliant behaviors. Thus, this study was conducted to investigate the mechanism of androstenone for changing horse behaviors. The change in the plasma concentrations of serotonin, β-endorphin, and cortisol in response to the treatment of androstenone was evaluated using an immunoassay. The results of this study demonstrated that androstenone may control the neuroendocrine system of horses, resulting in behavioral changes. This is the first work that studies the mechanism of pheromone treatment in horses and can be applied for further study about the effect of pheromone therapy on horses.

**Abstract:**

Androstenone influences the changing behaviors of animals. Previous studies discovered that an androstenone receptor was expressed in horses and treatment with androstenone induced horses to be more compliant. As changes in the level of neuroendocrine factors result in animal behavioral changes, the objective of the study was to monitor the changes in the concentrations of 5-HT, β-endorphin, and cortisol in response to androstenone. Eight thoroughbred horses (five mares and three geldings) were treated with androstenone diluted in jojoba oil (10 µg/mL) and only oil for a control cross-overly. A handler applied the treatments to the horses′ nostril and rubbed for 5 s. Blood samples were collected before, 15, 30, and 60 min after each treatment. The concentrations of each neurotransmitter were analyzed by enzyme-linked immunosorbent assay. The concentrations of each neurotransmitter after the treatment were compared to its baseline concentration. The concentration of 5-HT of the androstenone-treated horses remained consistent throughout the experiment, while the concentration of the control group significantly decreased over time. The plasma concentration of β-endorphin in the androstenone-treated group also remained constant, whereas the concentration increased in the control group. Cortisol levels did not change in either the treated or untreated groups. An androstenone treatment triggers changes in the secretion of 5-HT and β-endorphin in horses.

## 1. Introduction

Hormones and/or neurotransmitters act on the nervous system to regulate physiological responses and behavioral changes [1,2]. Several studies suggest that the synthesis and release of hormones and neurotransmitters are mediated by internal or external stimuli including pheromones [3,4,5]. Pheromones are volatile and non-volatile chemical molecules that usually trigger behavioral changes in the same species of animal [6]. 5α-androst-16-en-3-one (androstenone) is a pheromone contained in boar saliva [7]. We recently reported an expression of the androstenone specific receptor (OR7D4) in the vomeronasal organ and nasal cavity of horses [8], indicating that horses have an ability to sense androstenone. In addition, behavioral testing suggests that androstenone treatment induces docile behavior in horses (data not published).

Serotonin, also known as 5-hyxroxytryptamine (5-HT), is a monoamine neurotransmitter and is synthesized from tryptophan by the enzymes tryptophan, hydroxylase, and aromatic amino acid decarboxylase [9]. 5-HT is one of the factors that regulates and modulates the psychological state of animals [10,11]. 5-HT is also involved in the social behaviors of many animal species such as hamsters [9], rats [12], mice [13], dogs [14], and humans [15]. These studies show that high levels of 5-HT promote social interaction and decrease aggression in animals. Thus, the behavioral changes of horses, which see them become more compliant to handlers, caused by pheromone treatment may have a correlation with the 5-HT secretion. The results of previous studies led us to hypothesize that the concentration of plasma 5-HT could be induced by androstenone treatment.

β-endorphin is an opioid neuropeptide and peptide hormone secreted by certain neurons within the central nervous system (CNS) and peripheral system. β-endorphin functions to reduce stress and maintain homeostasis in the body. It generally modulates pain perception both in the central and the peripheral nervous systems. With the general characteristics of β-endorphin, the plasma β-endorphin may be induced by a series of blood collection stimuli to reduce stress. β-endorphin secretion also has a correlation with 5-HT secretion because the activation of the endogenous opioid system releasing β-endorphin is mediated by the serotonergic system [16,17]. Thus, we hypothesized that treatment with androstenone may regulate the β-endorphin secretion in the horses′ plasma.

Sport horses, especially racing or endurance horses, often have a high intensity of physical activity that leads to increased stress in the horses [18]. Thus, a treatment that can be used to reduce the stress of equine athletes should be developed because stress can be a limiting factor in the sporting ability of horses [19]. Cortisol is a steroid hormone in the glucocorticoid class and is produced mainly by the zona fasciculate of the adrenal cortex in the adrenal gland [20,21]. It is released by the hypothalamic-pituitary-adrenal axis (HPAA). Sustained stress can lead to high levels of circulating cortisol [22]. In the present study, the concentration of plasma cortisol was investigated to monitor the stress level of the horses during the experiment. The plasma cortisol level may increase due to the blood collection procedure. Thus, we hypothesized that treatment with androstenone may down-regulate cortisol secretion during the experimental procedure.

Based on the fact that animal behaviors can be determined by the status of neurotransmitters, we hypothesized that androstenone affects the social interaction between horses and humans via neurotransmitters such as serotonin, β-endorphin, or cortisol. The objective of this study was to monitor the changes in the concentrations of 5-HT, β-endorphin, and cortisol in response to androstenone. Thus, the change in the plasma concentrations of 5-HT, β-endorphin, and cortisol was sequentially monitored before and after treatment with androstenone.

## 2. Materials and Methods

### 2.1. Animals

This study was performed in May. Eight thoroughbred horses were used in this study, consisting of five barren mares and three geldings. The average age of the horses was 12 ± 1.7 years. All of the horses were used for horse riding lessons one or two times per day, except on a Monday. The horses were managed in the Sangju International Equestrian Center, Sangju, South Korea. The horses were stabled in private stalls and fed with 2% body weight (BW) timothy hay and 0.5% BW commercial concentrate. All of the horses had ad libitum access to water.

### 2.2. Ethical Note

The research protocol was approved by the Animal Experimentation Ethics Committee of Kyungpook National University (permit number: 2019-0177) and was performed according to the guidelines of the Animal Experimentation Ethics Committee. All of the researchers handled the horses cautiously to minimize the experiment time and stress level of the horses.

### 2.3. Pheromone Treatment

Androstenone (18339-16-7, Sigma-Aldrich, St. Louis, MO, USA) was diluted with jojoba oil (P18C035, Niceday365, Seoul, Korea) at a concentration of 10 µg/mL. A handler applied 2 mL of oil with or without androstenone to the horses′ nostril and rubbed for 5 s wearing latex gloves. For the negative control, jojoba oil without androstenone was used. Treatments with or without androstenone were applied to horses with a cross-over design.

### 2.4. Samples

Approximately 8 mL of blood was collected from the jugular vein before, 15, 30, and 60 min after treatment. Blood samples were collected in EDTA tubes (BD Vacutainer, Becton Drive Franklin Lakes, NJ, USA) and maintained in at 4 °C in an ice box during transportation. The plasma was separated from the whole blood using centrifugation at 1500× *g* for 10 min at 25 °C and stored in a −70 °C refrigerator before analysis.

### 2.5. Enzyme Linked Immunosorbent Assay (ELISA) for 5-HT

The 5-HT was analyzed with plasma using a horse 5-HT ELISA kit (MBS037450, MyBioSource, San Diego, CA, USA) following the manufacturer’s protocols. The sensitivity of the kit was 2.0 ng/mL. Analysis was performed with an undiluted sample in duplicate. A microplate reader (Tecan, Männedorf, Switzerland) calculated the concentration of 5-HT in the sample based on an absorbance of a wavelength of 450 nm. The average intra- and inter-assay coefficients of variation were 15 and 15%, respectively.

### 2.6. ELISA for β-Endorphin

The β-endorphin was analyzed with plasma using a horse Bep (β-endorphin) ELISA kit (IT1465, Immunotag^TM^, St. Louis, MO, USA) following the manufacturer’s protocols. The sensitivity of the kit was less than 9.375 pg/mL. Samples were diluted with a sample dilutant at a ratio of 1:2 and analyzed in duplicate. A microplate reader (Tecan, Switzerland) calculated the concentration of β-endorphin in the sample based on an absorbance of a wavelength of 450 nm. The average intra and inter assay coefficients of variation were less than 8 and 10%, respectively.

### 2.7. ELISA for Cortisol

The cortisol was analyzed with plasma using a Cortisol ELISA kit (ADI-900-071, Enzo Life Science, New York, NY, USA) following the manufacturer’s protocols. The sensitivity of the kit was 56.72 pg/mL. Samples were diluted with a steroid displacement reagent at a ratio of 1:100 and analyzed in duplicate. A microplate reader (Tecan, Switzerland) calculated the concentration of cortisol, 5-HT, and β-endorphin in the samples based on an absorbance of a wavelength of 405 nm. The average intra- and inter-assay coefficients of variation were less than 10.5 and 13.4%, respectively.

### 2.8. Statistical Anlalysis

The interaction effect of each treatment of androstenone on the level of neurotransmitters was assessed using a Mixed GLM model and the SAS program (SAS Institute, Cary, NC, USA). A least square (LS) post-hoc test was applied to test the significant difference in the plasma concentration of neurotransmitters between the treatment and the cortisol group at the same sampling time. Time-dependent hormonal changes were also compared using a LS means comparison. The *p* values of <0.05 were considered to be statistically significant (*p* < 0.05).

## 3. Results

### 3.1. 5-HT Concentration According to the Time of Androstenone Treatment

Thirty minutes after the control treatment, the plasma 5-HT levels were significantly reduced compared to baseline levels (Table 1). At 15 and 60 min, the 5-HT levels were similar to the initial concentrations in the control group. Interestingly, the 5-HT concentrations of the androstenone-treated animals did not change at 15, 30, or 60 min compared to the baseline.

### 3.2. β-Endorphin Concentration According to the Time of Androstenone Treatment

The concentration of β-endorphin at 15 and 30 min after oil-only treatment significantly increased compared to the sample collected before the treatment (Table 1). However, the concentration of β-endorphin at 60 min after the oil treatment showed no significant change. Unlike the results of the control group, there was no change in the levels of plasma β-endorphin in the androstenone-treated animals at 15, 30, and 60 min after treatment, compared to the baseline concentration.

### 3.3. Cortisol Concentration According to the time of Androstenone Treatment

The cortisol concentrations remained unchanged throughout the duration of testing in the control group and in the androstenone-treated group (Table 1).

## 4. Discussion

Several studies have demonstrated that pheromones elicit changes in the endocrine system [23,24]. In the present study, the plasma concentration of 5-HT in the control group significantly decreased 30 min after oil treatment, while the treatment group maintained similar levels of 5-HT concentration during the experiment. 5-HT is one of the key factors that regulate and modulate the psychological state of animals [10,11], and the role is important in animal behavior involving anxiety [25,26]. The minor pain caused by the repeated blood collection may have induced uneasiness in the horses, which may have contributed to the decrease in the plasma 5-HT concentration. 5-HT is involved in systems that are able to modulate the transmission and processing of nociceptive information [27]. In the treatment group, the plasma concentration of 5-HT did not change during the experiment, which suggests that the perception of pain due to the repeated blood collection was attenuated. Thus, it is possible that androstenone treatment facilitated social interactions between the experimenter and the horses, and reduced the discomfort associated with blood collection. This explanation can be supported by our previous data demonstrating that androstenone treatment on horses encouraged the interaction between handlers and horses (data not published). Several studies support the suggestion that social interaction reduces the perception of pain in human subjects [28,29,30]. Consequentially, the androstenone treatment seemed to increase the pain threshold of the horses through continuing 5-HT secretion.

β-endorphin is an opioid neuropeptide that functions to reduce stress and maintain homeostasis in the body, and generally modulates pain perception both in the central and the peripheral nervous systems [31]. In horses, plasma β-endorphin can be used as an indicator of stress and pain [32]. McCarthy et al. suggested that the application of an upper lip twitch to horses results in a dramatic increase in the plasma β-endorphin concentration. Similarly, in our study, we found that β-endorphin levels were elevated at the 15- and 30-minute blood collections in the control group, indicating that repeated blood collection causes enough pain to release β-endorphins. Interestingly, the concentration of β-endorphins in the treatment group showed no significant change during the series of blood collections. Endorphins inhibit the pain signaling pathway [33]. Thus, the androstenone had an analgesic effect that increased the pain threshold of the horses. The activation of the endogenous opioid system that released β-endorphins is mediated by the serotonergic system [16,17]. The result of the β-endorphin concentration in response to the androstenone treatment may be associated with the maintenance of 5-HT levels and the social buffering of pain perception.

Plasma cortisol is produced when the HPAA is activated by stressors, and the concentration of plasma cortisol has been used as a marker for determining a stress response [34]. In the case of mice, single or multiple injections moderately increased the plasma corticosterone concentrations [35]. The authors suggested that handling or injection could be stressors affecting the physiological parameters [35]. We hypothesized that the series of blood collections using a syringe might cause a stress response in the horses, inducing a plasma cortisol increase. In addition, we expected the androstenone treatment to down-regulate cortisol secretion. However, we found that there was no significant change in the concentration of cortisol in both the treatment and control group. In a study of race horses, cortisol levels during venous catheter placement did not differ significantly from the baseline [36]. This means that venipuncture should not involve high stress to horses. The most effective factors for activating the HPAA system that induces increasing glucocorticoids are generally fearfulness, which is derived from unexpected environmental challenges that are threatening mental or bodily stability in horses [37]. Thus, the repeated blood collection during the study was not as critical as to increase the plasma cortisol concentration. Further study is needed to determine whether androstenone treatment has an effect on plasma cortisol in response to moderate or intense stress.

## 5. Conclusions

Androstenone treatment triggers a constant secretion of 5-HT in horses that sees them become more tolerant of discomfort. Thus, androstenone could be used as an effective aid to calm horses.

## Figures and Tables

**Table 1:** Time dependent plasma 5-HT, β-endorphin, and cortisol concentrations of androstenone-treated and untreated groups.

	Control (Oil)				Treatment (Androstenone)			
	Before	15 min	30 min	60 min	Before	15 min	30 min	60 min
5-HT (ng/mL)	98.6 ± 18.3 ^a^	92.3 ± 16.5^ab^	90.2 ± 17.2	93.8 ± 17.9	94.6 ± 18.2	94.1 ± 17.4	91.6 ± 17.7	89.5 ± 17.0
β-endorphin (pg/mL)	233.6 ± 30.0	265.7 ± 33.7^ab^	282.2 ± 35.5 ^b^	287.1 ± 39.5 ^b^	300.8 ± 52.3	302.82 ± 52.4	282.1 ± 40.9	303.2 ± 54.6
Cortisol (ng/mL)	4.9 ± 1.5	6.0 ± 0.9	6.5 ± 1.1	4.9 ± 0.7	4.8 ± 0.9	6.2 ± 1.1	6.2 ± 1.3	5.2 ± 0.9

^a, b^ Different superscripts indicate statistical significance (*p* < 0.05).

## Data Availability

The data presented in this study are available within the article.
